# Effects of Polysaccharides from *Platycodon grandiflorum* on Immunity-Enhancing Activity In Vitro

**DOI:** 10.3390/molecules22111918

**Published:** 2017-11-07

**Authors:** Xiaona Zhao, Yuge Wang, Peng Yan, Guodong Cheng, Cheng Wang, Na Geng, Xuepeng Wang, Jianzhu Liu

**Affiliations:** 1 College of Animal Medicine and Veterinary Medicine, Shandong Agricultural University, Tai’an 271018, China; zhaoxn@sdau.edu.cn (X.Z.); 18354808000@163.com (P.Y.); 18764880103@163.com (G.C.); vetliujz@163.com (C.W.); gengna1440@163.com (N.G.); 2 Research Center for Animal Disease Control Engineering Shandong Province, Shandong Agricultural University, Tai’an 271018, China; wangyuge0323@163.com; 3 Shandong Provincial Engineering Technology Research Center of Animal Disease Control and Prevention, Shandong Agricultural University, 61 Daizong Street, Tai’an 271018, China

**Keywords:** *Platycodon grandiflorum*, polysaccharides, lymphocyte proliferation, cell cycle, immunity-enhancing

## Abstract

The study is aimed at investigating the immunoenhancement activity of polysaccharides from *Platycodon grandiflorum* polysaccharides (PGPSs) in vitro. In this study, some research on lymphocyte proliferation, cell cycle, and the levels of CD4^+^ and CD8^+^ T cells were performed. Four different concentrations of PGPSs (PGPS_tc_, PGPS_60c_, PGPS_80c_, and PGPS_tp_) were harvested and added to peripheral blood T lymphocytes. We observed significant increases in T lymphocyte proliferation at PGPS_tc_ groups individually or synergistically with phytohemagglutinin (PHA) at most concentrations, and their lymphocyte proliferation rates were the highest. The active sites of PGPS_tc_ and PGPS_60c_ were subsequently chosen. Then, we utilized flow cytometry to determine lymphocyte cell cycle distribution and levels of CD4^+^ and CD8^+^ T cells. At most time points, PGPS_tc_ could facilitate lymphocyte cell cycle progression from the G0/G1 phase to the S and G2/M phases and, simultaneously, increase the levels of CD4^+^ and CD8^+^ T cells. These results indicate that PGPS_tc_ enhances the immune functions, suggesting that PGPS_tc_ could be a potential immunopotentiator for further in vivo and clinical trial experiments.

## 1. Introduction

Bioactive compounds from marine organisms with antimicrobial activity have been ascribed to a variety of metabolites, such as polysaccharides [1,2]. Polysaccharides with a low risk of side effects and toxicity, which can activate immune cells, improve immune function and have been found in normal cells. Interest in polysaccharides as new immunopotentiators for the development of veterinary vaccines has recently increased. Some studies have demonstrated that *Astragalus* and *Echinacea* polysaccharides have potential for use as immunopotentiators/adjuvants in inactivated rabies vaccines for veterinary use [3]. Taishan *Robinia pseudoacacia* polysaccharide can improve immunologic function and be used as a vaccine immunopotentiator for immune responses [4]. *Astragalus* polysaccharide (APS) and sulfated APS (SAPS) show dose-dependent growth-facilitating and immunomodulating function, and could be a potential trigger for immunomodulator in early LPS stimulation condition [5].

*Platycodon grandiflorum* (PG) has been used as either a food material as well as in traditional Chinese medicine to prevent or treat a variety of diseases, such as for treating cough, phlegm, hyperlipidemia, hypertension, diabetes and immunoregulation [6]. Polysaccharides extracted from PG had been shown to activate various cell types of the innate and adaptive immune systems. It is evident that PG polysaccharides induce DC maturation by activating MAPK and NF-κB signaling downstream of TLR4, and they might be used as adjuvants in DC-based cancer immunotherapy [7]. Moreover, another study has shown that MAPK/AP-1 and TLR4/NF-κB signaling pathways were involved in the macrophage activation by PG polysaccharides (PGPSs) [8].

In the present study, four types of polysaccharides, PGPS_tc_, PGPS_60c_, PGPS_80c_, and PGPS_tp_, were extracted from PG and purified. We investigated peripheral T lymphocyte proliferation activity at the cellular level in immune system, and further studies were examined on distributions of cell cycles and percentages of CD4^+^ and CD8^+^ T cells. Our research shows that PGPS_tc_ had a significant impact on immunoenhancement activity and could be used as a novel immunopotentiator to develop.

## 2. Materials and Methods

### 2.1. Reagents

RPMI-1640 media (Gibco, Grand Island, NY, USA) filtered (0.22 μm) and added with benzylpenicillin (100 IU·mL^−1^), streptomycin (100 IU·mL^−1^), and fetal bovine serum (10%) was utilized to culture cells. Phytohemagglutinin (PHA-P, Sigma, St. Louis, MO, USA) was dissolved into 0.5 mg·mL^−1^ with RPMI-1640 media. 3-(4,5)-Dimethylthiazol-2,5-diphenyltetrazolium bromide (MTT) was dissolved with PBS, and the final concentration was 5 mg·mL^−1^. Dimethyl sulfoxide (purchased from Solarbio Technology Co., Ltd., Beijing, China). Lymphocyte separation medium (purchased from Tianjin Haoyang Biological Manufacturing Co., Ltd., Tianjin, China). A PHA solution was placed at −20 °C, and an MTT solution was stored at 4 °C for 2 weeks without light.

The use and care of Hy-line adult Cocks (male, 60-day old) were from the Experimental Animal Center of the Shan Dong Agricultural University and were approved by the College Committee for animal experiments (Permit number: SDAUA-2014-012).

### 2.2. Preparation of PGPSs

The dried rhizome of PG was acquired from Jin Tai Lian Co. Ltd., Tai’an, China. Adopting alcohol sedimentation, three crude polysaccharides, crude total PG (PGPS_tc_), and fractional PG polysaccharides (PGPS_60c_ and PGPS_80c_) were prepared in our laboratory. Briefly, PGPSs were obtained via water extraction and alcohol precipitation methods, and the final concentration was 1 g·mL^−1^. PGPS_tc_ was extracted by one-step ethanol precipitation, in which ethanol was added to the decoction to obtain ethanol concentration of 80% (*v*/*v*). Two fractional polysaccharides (PGPS_60c_ and PGPS_80c_) were extracted via stepwise ethanol precipitation with ethanol concentration at 60% and 80%, respectively.

The purified PGPS_tp_ was obtained using Sevag’s method to eliminate protein [9], through a Sephadex G-75 column and eluting with distilled water. The eluent in a dialysis sack was dialyzed against flowing distilled water for 12 h. Then, the dialysate was lyophilized, and the powder of purified PGPS_tp_ was collected. The carbohydrate contents of PGPS_tc_, PGPS_60c_, PGPS_80c_ and PGPS_tp_ were 64%, 53.7%, 63.5% and 73%, respectively, as measured by a phenol hydrate-sulfuric acid method using analytical grade glucose as a standard sample [10]. The structure identification of PGPSs was assessed in our previous study [11].

### 2.3. Peripheral Lymphocyte Proliferation Assay

The MTT assay was performed to measure peripheral lymphocyte proliferation as described in our previous study [12]. The absorbance of cells in each well was read at 570 nm using a microplate reader (DG-3022, Nanjing Huadong Electronics, Information & Technology Co., Ltd., Nanjing China).

The highest proliferation rate was evaluated: the highest proliferation rate = (the highest OD_570_ value of the experimental group—the OD_570_ value of the control group or the PHA group)/(the OD_570_ value of the control group or the PHA group) × 100%. Then, PGPS_tc_ and PGPS_60c_ with better activity were subsequently selected.

### 2.4. Cell Cycle Distribution Analysis

Cell cycle was analyzed by flow cytometer (BD Biosciences FACSCanto II Flow Cytometer, BioLegend, San Diego, CA, USA) [13]. The lymphocytes as described above were challenged with PGPS_60c_ and PGPS_tc_ (31.25 μg·mL^−1^, selected according to the preliminary experimental results) at 37 °C, 5% CO_2_. After 24, 48, and 72 h treatments, the lymphocyte cells were collected, fixed with 70% cold ethanol, and left overnight. After washing twice with cold PBS, the lymphocyte cells were labeled with propidium iodide (PI) solution (50 μg·mL^−1^) in the presence of RNase A (500 μg·mL^−1^) and incubated at 37 °C without light for 30 min. The distribution of cells in G0/G1, S, and G2/M phases were analyzed via ModFit LT software (FACS Calibur™, Becton-Dickinson, Franklin Lakes, NJ, USA). The proliferation index was evaluated as SPF and PI values according to the following formula [14]. SPF = S/(G0/G1 + S + G2/M) × 100%. PI = (S + G2/M)/(G0/G1 + S + G2/M) × 100%.

### 2.5. Detection of CD4^+^ and CD8^+^ T Lymphocytes

The expressions of CD4^+^ and CD8^+^ on lymphocytes were determined with a flow cytometer (BD Biosciences FACSCanto II Flow Cytometer, BioLegend, San Diego, CA, USA). After 24, 48, and 72 h interference by PGPSs, the cells were collected and washed twice with cold PBS. Then, 100 μL of PBS were added to resuspended. Then, the cells were stained with 10 μL of anti-CD4-FITC and 10 μL of anti-CD8-PE, and the mixture was incubated at 37 °C without light for 30 min. Finally, the stained cells were washed with PBS and separated at 1000 rpm for 10 min [15].

### 2.6. Statistical Analysis

SPSS 16.0 (SPSS Inc., Chicago, IL, USA) was used for statistical analysis with one-way ANOVA. Multiple comparisons among the control and polysaccharide groups were conducted. All experiments were repeated at least thrice. Data were presented as means ± standard errors (SE). *p*-Values of differences less than 0.05 were considered significant.

## 3. Results

### 3.1. Characterization of Platycodon grandiflorum

The structure identification of PGPSs was assessed in our previous research [11]. FT-IR analysis showed that PGPSs possess typical absorption peak of polysaccharides. Molecular weight distribution showed that all PGPSs had two peaks, at two different retention times. PGPSs were primarily composed of glucose, mannose, arabinose, and galactose, and they were linked mainly by (1→3,6)-β-d-Galp residues.

### 3.2. Cytotoxicity of PGPSs to Peripheral Lymphocytes

The OD_570_ values of each group are presented in Table 1. The OD_570_ values of PGPS_60c_ at 250 μg·mL^−1^, PGPS_80c_ at 1000 μg·mL^−1^, PGPS_tc_ at 500 μg·mL^−1^, and PGPS_tp_ at 62.5 μg·mL^−1^ were just not lower than those of the control group significantly (*p* > 0.05). Therefore, the corresponding concentration could be reckoned as the safety concentration, respectively. For the convenience of comparison of PGPSs at the same level, the safety concentrations of PGPSs were unified as 62.5 μg·mL^−1^.

### 3.3. Peripheral Lymphocyte Proliferation in Single Stimulation with PGPSs

Table 2 indicates the changes of OD_570_ values of each group. The OD_570_ values of PGPS_60c_, PGPS_80c_, and PGPS_tc_ at 3.907~62.5 μg·mL^−1^ and of PGPS_tp_ at 15.625~62.5 μg·mL^−1^ were significantly higher than those of the control group (*p* < 0.05).

**Figure 1:**
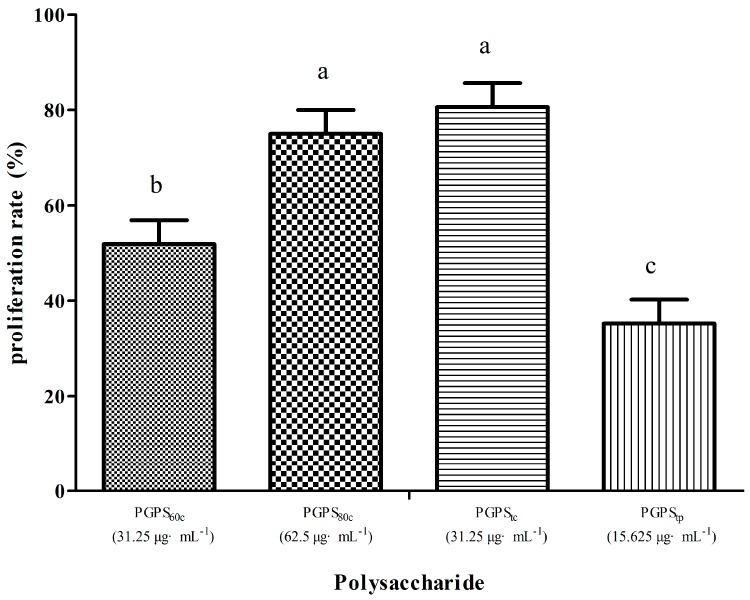
The highest lymphocyte proliferation rate of each group in single stimulation with PGPSs. The values are presented as means ± SE (*n* = 4). ^a–c^ Bars without the same superscripts differ significantly (*p <* 0.05).

Figure 1 shows the highest lymphocyte proliferation rate. In a single stimulation with polysaccharides, the proliferation rate in PGPS_tc_ at 31.25 μg·mL^−1^ was the highest (80.61%), followed by PGPS_80c_ at 62.5 μg·mL^−1^ (75%). The proliferation rates in the PGPS_tc_ and PGPS_80c_ groups were likewise significantly higher than those in the PGPS_60c_ and PGPS_tp_ groups (*p* < 0.05).

### 3.4. Peripheral Lymphocyte Proliferation in Synergistic Stimulation of PGPSs with PHA

Table 3 indicates the changes in OD_570_ values of each group. The OD_570_ values of PGPS_60c_ at 15.625~31.25 μg·mL^−1^, of PGPS_tc_ at 3.907~31.25 μg·mL^−1^, and PGPS_tp_ at 3.907~15.625 μg·mL^−1^ were significantly higher (*p* < 0.05) than those in the control group (*p* < 0.05).

**Figure 2:**
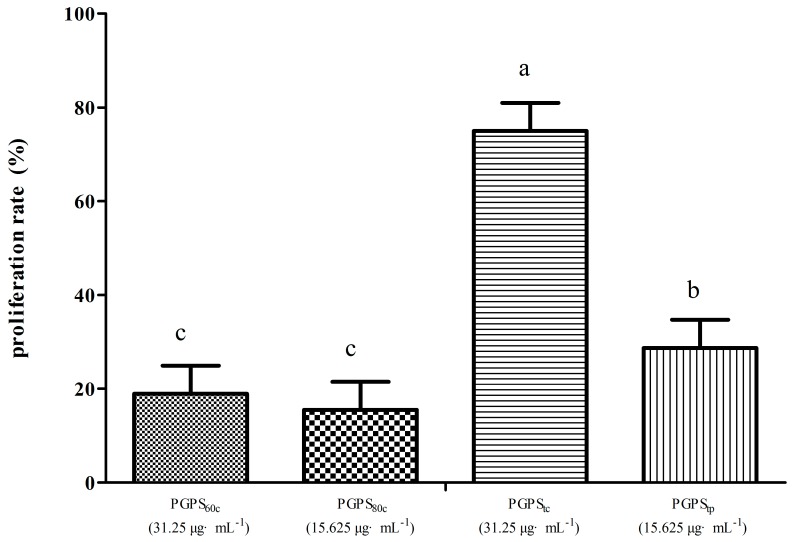
The highest lymphocyte proliferation rates of each group in synergistically stimulation of PGPSs with PHA. The values are presented as means ± SE (*n* = 4). ^a–c^ Bars without the same superscripts differ significantly (*p <* 0.05).

Figure 2 illustrates the highest lymphocyte proliferation rate of four groups. The proliferation rate in PGPS_tc_ at 31.25 μg·mL^−1^ was the highest (75%), followed by PGPS_tp_ at 7.813 μg·mL^−1^ (40%). The proliferation rate of cells in PGPS_tc_ was considerably higher than those in PGPS_60c_, PGPS_80c_, and PGPS_tp_ (*p* < 0.05).

### 3.5. Cell Cycle Analysis

**Figure 3:**
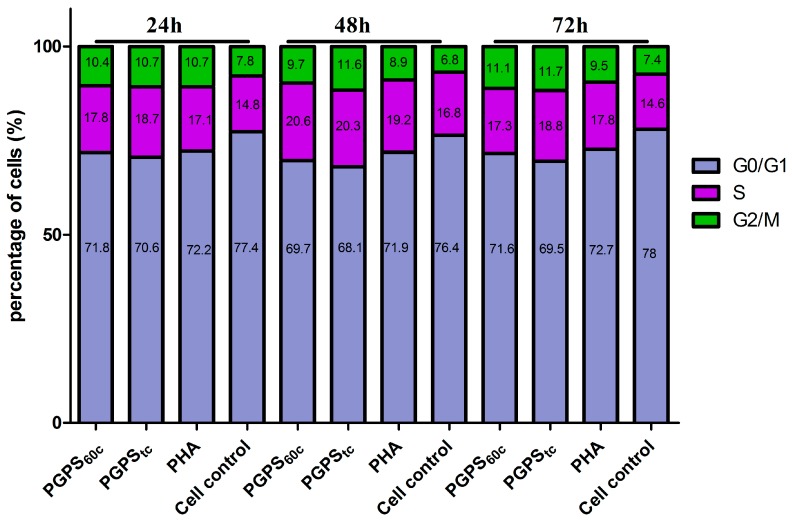
Changes of cell cycle distribution in synergistically stimulation of PGPSs with PHA. The values are presented as means ± SE (*n* = 4).

As shown in Figure 3, compared to cell control group, PHA induced a notable decrease in the percent of cells in the G0/G1 phase (*p* < 0.05) but a significant increase of cells in the S and G2/M phases at all the time points (*p* < 0.05). The ratio of cells decreased in the G0/G1 phase and increased in the S phase in the PGPS_tc_ group over 24 h after incubation (*p* < 0.05), and the percentage of cells in the G2/M phase had no significant change after treatment for 24 h. However, after treatment with PGPS_tc_ for 48 h, there was a significant reduction in the amount of cells in the G0/G1 phase but an increase in the S and G2/M phases (*p* < 0.05) which did not significantly change compared with the PHA group. The same in the G2/M phase as the PGPS_60c_ group. Treated with PGPS_tc_ for 72 h, a significant reduction occurred in the percentage of cells in the G0/G1 phase, whereas there was an increase in the G2/M phases (*p* < 0.05); however, there were no notable differences when compared with the PHA group. At most time points, SPF and PI in PGPS_tc_ were the largest among all groups (Table 4 and Table 5).

### 3.6. Changes in Lymphocytes CD4^+^ and CD8^+^ T Cells

**Figure 4:**
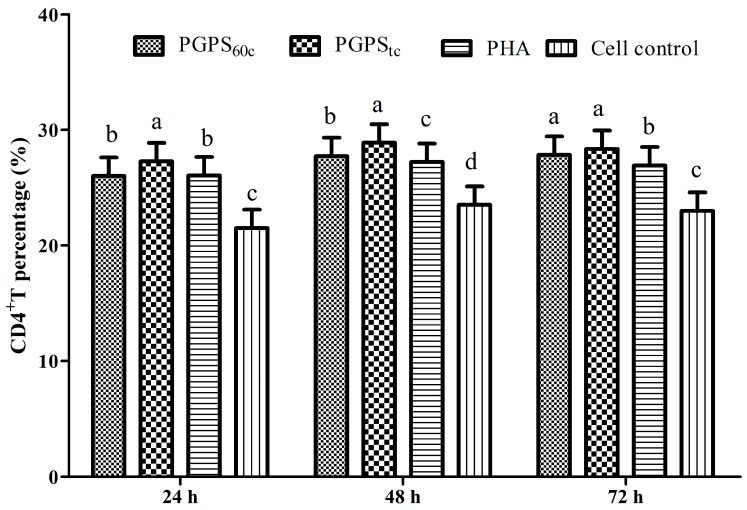
Changes of peripheral blood CD4^+^ T cells in stimulation of PGPSs or PHA. The values are presented as means ± SE (*n* = 4). ^a–d^ Bars without the same superscripts differ significantly (*p <* 0.05).

The changes of CD4^+^ T cells are presented in Figure 4. The percentages of CD4^+^ T cells in PGPS_tc_ at 24, 48, and 72 h, and in PGPS_60c_ at 48 h and 72 h, were markedly higher than those in the PHA group (*p* < 0.05). At the time points of 48 h and 72 h, the proportion of CD4^+^ T cells in the PGPS_tc_ group were markedly higher than those in the PGPS_60c_ group (*p* < 0.05).

**Figure 5:**
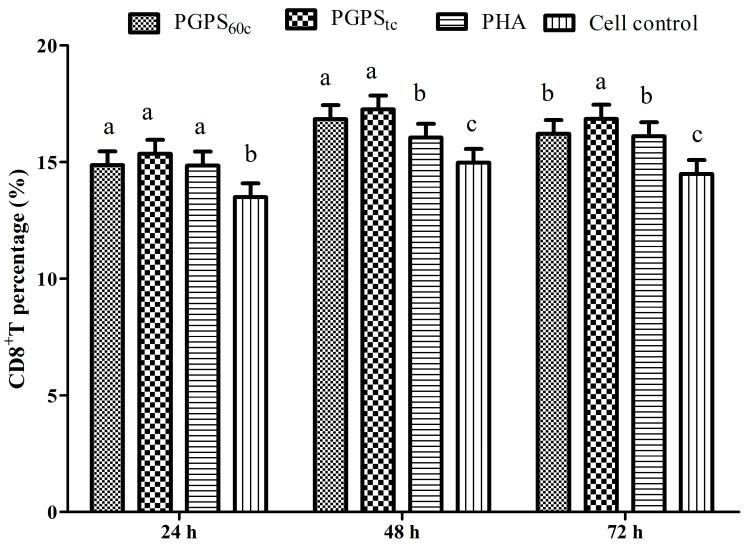
Changes of peripheral blood CD8^+^ T cells in stimulation of PGPSs or PHA. The values are presented as means ± SE (*n* = 4). ^a–c^ Bars without the same superscripts differ significantly (*p <* 0.05).

The changes in CD8^+^ T cells are presented in Figure 5. At 48 h and 72 h, the proportion of CD8^+^ T cells in the PGPS_tc_ group was markedly higher than those in the PHA group (*p* < 0.05). At 72 h, the proportion of CD8^+^ T cells in the PGPS_tc_ group was markedly higher than those in the PGPS_60c_ group (*p* < 0.05).

At 72 h, the proportion of CD8^+^ T cells in the PGPS_tc_ group was observably higher than those in the PGPS_60c_ group (*p* < 0.05).

**Figure 6:**
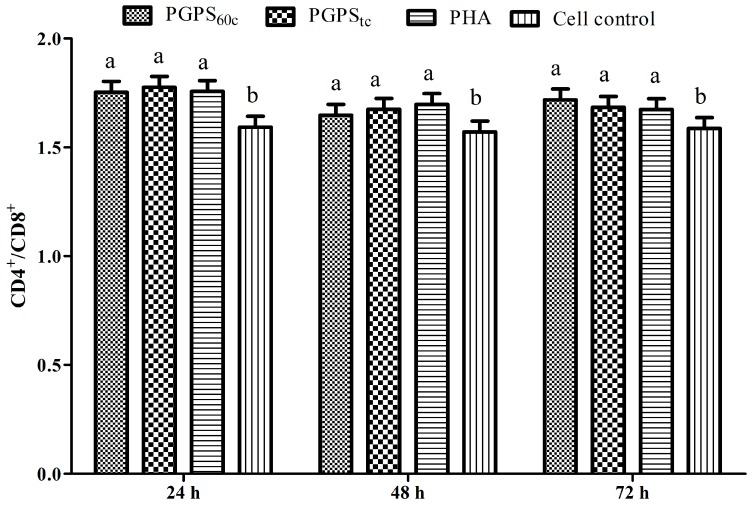
Changes of peripheral blood CD4^+^/CD8^+^ of polysaccharides. The values are presented as means ± SE (*n* = 4). ^a–b^ Bars without the same superscripts differ significantly (*p* < 0.05).

The changes in CD4^+^/CD8^+^ ratios are presented in Figure 6. At three time points, the CD4^+^/CD8^+^ ratios in the PGPS_tc_ and PGPS_60c_ groups were significantly higher than that in the cell control group (*p* < 0.05), which indicated that the polysaccharides increased the ratio of CD4^+^/CD8^+^.

## 4. Discussion

T lymphocyte, which can be induced by PHA, is responsible for cell-mediated immunity [16,17]. By means of accelerating the clearance of pathogens and producing immunomodulatory cytokines, cellular immunity responses play a critical role in the host defense system against infections [18]. In this study, it was observed that PGPS_60c_, PGPS_80c_, PGPS_tc_ (3.907~62.5 μg·mL^−1^), and PGPS_tp_ (15.625~62.5 μg·mL^−1^) alone increased the OD_570_ values, the highest proliferation rate of cells was presented in PGPS_tc_, followed by PGPS_80c_, suggesting that they promoted cell proliferation of the lymphocytes. Besides, PGPS_60c_ (15.625~31.25 μg·mL^−1^), PGPS_tc_ (3.907~31.25 μg·mL^−1^), and PGPS_tp_ (3.907~15.625 μg·mL^−1^) also enhanced PHA-induced OD_570_ values, and the highest proliferation rate of cells was presented in PGPS_tc_, followed by PGPS_tp_, which implied that they synergized with PHA in promoting proliferation of the lymphocytes. Thus, the active sites of PGPS_tc_ and PGPS_60c_ were selected and subjected to further experiments by synthetic analysis. Zhang et al. have demonstrated that in vitro *Ganoderma lucidum* polysaccharide (GLP) could significantly enhance lymphocytes proliferation singly or synergistically with ConA [19]. Liu et al. have reported that *Atractylodes macrocephala* polysaccharide (AMP) and its selenium-modified products at certain concentrations could significantly promote lymphocytes proliferation and enhance cellular immunity [20].

CD4^+^ is a T helper (Th), while CD8^+^ is a Tcytotoxic (Tc) lymphocyte, and these are two common T lymphocytes vital for adaptive immunity [21]. Some researchers have reported that the percentage of CD4^+^ or CD8^+^ cells and the ratio of CD4^+^/CD8^+^ increased when treated with ginseng fruit polysaccharides (CMPs) substantially compared with the negative control group, which is in line with the literature [22]. The effect *of Jujube polysaccharide* conjugates (JPCs) treatment was better than *Ginsenoside* treatment on the growth of counts of CD4^+^ T cell and the ratio of CD4^+^/CD8^+^ [23]. These results were consistent with our data, which proved the cellular and humoral immune functions. In this study, PGPS_tc_ and PGPS_60c_ also improved the proportion of CD4^+^ and CD8^+^ T cells effectively, and PGPS_tc_ produced optimal effects.

The cell cycle refers to a series of processes that take place in a cell to allow division and duplication period. Research showed that polysaccharide isolated from Yu-Ping-Feng (YPF-PS) could significantly increase lymphocyte cells entering into the S and G2/M phases at most time points by the in vitro and in vivo tests [24]. Previous reports have shown that *Atractylodis macrocephalae Koidz* polysaccharides (RAMPS_60c_ and RAMPS_tp_) could promote cells into the S and G2/M phases [14]. This result was consistent with the present data, which indicated that they can regulate the cell cycle progression. In this study, the results clearly showed that PGPS_tc_ could accelerate the cell cycle and promote lymphocytes proliferation, which underlines the importance of PGPS_tc_ in these processes. This is supported by other related research [25]. These results showed that the immune-enhancing activity of PGPS_tc_ may be related to its ability which can stimulate the proliferation of lymphocytes.

## 5. Conclusions

Polysaccharides from *Platycodon grandiflorum* (PGPS) exhibited the immunoenhancement activity in vitro. Moreover, PGPS_tc_ could significantly promote the proliferation of lymphocyte individually or synergistically with PHA-P at most concentrations, facilitate lymphocyte cell cycle progression from the G0/G1 phase to the S and G2/M phases, and increase the levels of CD4^+^ and CD8^+^ T cells, which indicated that PGPS_tc_ could improve immune functions. Therefore, PGPS_tc_ might be helpful for developing a novel immunopotentiator. Further studies of PGPS are under investigation.

## Figures and Tables

**Table 1:** OD_570_ value of each polysaccharide group within 2000–1.953 μg·mL^−1^ (*n* = 4).

Concentration (μg·mL^−1^)	PGPS_60c_	PGPS_80c_	PGPS_tc_	PGPS_tp_
2000	0.111 ± 0.003 ^c^	0.101 ± 0.004 ^c^	0.113 ± 0.001 ^d^	0.106 ± 0.002 ^f^
1000	0.110 ± 0.010 ^c^	0.120 ± 0.002 ^b^	0.114 ± 0.001 ^c,d^	0.109 ± 0.003 ^e,f^
500	0.141 ± 0.006 ^a^	0.125 ± 0.005 ^c^	0.118 ± 0.002 ^b,c,d^	0.110 ± 0.003 ^d,e,f^
250	0.131 ± 0.009 ^a,b^	0.128 ± 0.002 ^b^	0.118 ± 0.001 ^b,c,d^	0.112 ± 0.003 ^c,d,e,f^
125	0.130 ± 0.002 ^a,b^	0.127 ± 0.003 ^b^	0.118 ± 0.002 ^b,c,d^	0.114 ± 0.001 ^c,d,e^
62.5	0.126 ± 0.004 ^b^	0.179 ± 0.002 ^a^	0.119 ± 0.002 ^b,c,d^	0.130 ± 0.003 ^a^
31.25	0.128 ± 0.001 ^a,b^	0.125 ± 0.002 ^b^	0.133 ± 0.002 ^a^	0.130 ± 0.001 ^a^
15.625	0.128 ± 0.004 ^a,b^	0.126 ± 0.006 ^b^	0.133 ± 0.001 ^a^	0.130 ± 0.002 ^a^
7.813	0.126 ± 0.001 ^b^	0.125 ± 0.001 ^b^	0.138 ± 0.001 ^a^	0.123 ± 0.003 ^a,b^
3.907	0.131 ± 0.003 ^a,b^	0.126 ± 0.000 ^b^	0.121 ± 0.004 ^b,c^	0.118 ± 0.001 ^b,c^
1.953	0.128 ± 0.003 ^a,b^	0.128 ± 0.001 ^b^	0.119 ± 0.004 ^b,c,d^	0.117 ± 0.002 ^b,c,d^
Cell control	0.125 ± 0.002 ^b^	0.125 ± 0.002 ^b^	0.124 ± 0.001 ^b^	0.124 ± 0.001 ^a,b^

^a–f^ Data within a column without the same superscripts differ significantly (*p <* 0.05).

**Table 2:** Changes in OD_570_ value of peripheral lymphocyte proliferation of each group in single stimulation with *Platycodon grandiflorum* polysaccharides (PGPSs) (OD_570_ value) (*n* = 4).

Concentration (μg·mL^−1^)	PGPS_60c_	PGPS_80c_	PGPS_tc_	PGPS_tp_
62.5	0.196 ± 0.009 ^a^	0.224 ± 0.019 ^a^	0.210 ± 0.007 ^a^	0.151 ± 0.005 ^a^
31.25	0.202 ± 0.005 ^a^	0.219 ± 0.009 ^a^	0.224 ± 0.024 ^a^	0.160 ± 0.013 ^a^
15.625	0.200 ± 0.004 ^a^	0.208 ± 0.010 ^a,b^	0.219 ± 0.012 ^a^	0.165 ± 0.009 ^a^
7.813	0.192 ± 0.003 ^a^	0.200 ± 0.010 ^a,b^	0.213 ± 0.007 ^a^	0.162 ± 0.008 ^a,b^
3.907	0.190 ± 0.015 ^a^	0.182 ± 0.006 ^b^	0.218 ± 0.007 ^a^	0.168 ± 0.003 ^a,b^
Cell control	0.133 ± 0.006 ^b^	0.128 ± 0.0039 ^c^	0.122 ± 0.004 ^b^	0.122 ± 0.004 ^b^

^a–c^ Data within a column without the same superscripts differ significantly *(p <* 0.05*).*

**Table 3:** Changes in OD_570_ value of peripheral blood lymphocyte proliferation of each group in synergistically stimulation of PGPSs with phytohemagglutinin (PHA) (OD_570_ value) (*n* = 4).

Concentration (μg·mL^−1^)	PGPS_60c_	PGPS_80c_	PGPS_tc_	PGPS_tp_
62.5	0.122 ± 0.002 ^a^^,b,c^	0.123 ± 0.004 ^a^	0.152 ± 0.029 ^a,b^	0.135 ± 0.009 ^b,c^
31.25	0.132 ± 0.004 ^a^	0.118 ± 0.004 ^a^	0.210 ± 0.008 ^a^	0.130 ± 0.010 ^b,c^
15.625	0.125 ± 0.002 ^a,b^	0.126 ± 0.006 ^a^	0.187 ± 0.002 ^a^	0.148 ± 0.009 ^a,b^
7.813	0.113 ± 0.000 ^b,c^	0.125 ± 0.006 ^a^	0.184 ± 0.005 ^a^	0.161 ± 0.013 ^a^
3.907	0.115 ± 0.002 ^b,c^	0.118 ± 0.005 ^a^	0.186 ± 0.007 ^a^	0.145 ± 0.007 ^a,b^
PHA	0.111 ± 0.003 ^c^	0.112 ± 0.004 ^a^	0.120 ± 0.002 ^b^	0.115 ± 0.002 ^c^

^a^^–c^ Data within a column without the same superscripts differ significantly *(p* < 0.05*).*

**Table 4:** SPF value of peripheral lymphocyte cell cycle of each group at different time points (*n* = 4).

Group	SPF		
	24 h	48 h	72 h
PGPS_60c_	17.787 ± 0.202 ^a,b^	20.630 ± 0.052 ^a^	17.277 ± 0.993 ^b^
PGPS_tc_	18.673 ± 0.511 ^a^	20.263 ± 0.265 ^a^	18.800 ± 0.779 ^a^
PHA	17.050 ± 0.309 ^b^	19.203 ± 0.178 ^b^	17.767 ± 0.393 ^a,b^
Cell control	14.830 ± 0.432 ^c^	16.760 ± 0.382 ^c^	14.613 ± 0.296 ^c^

^a–c^ Data within a column without the same superscripts differ significantly (*p* < 0.05).

**Table 5:** PI value of peripheral lymphocyte cell cycle of each group at different time points (*n* = 4).

Group	PI		
	24 h	48 h	72 h
PGPS_60c_	28.223 ± 0.419 ^b^	30.330 ± 0.427 ^b^	28.393 ± 0.435 ^a,b^
PGPS_tc_	29.347 ± 0.077 ^a^	31.860 ± 0.639 ^a^	30.490 ± 0.524 ^a^
PHA	27.713 ± 0.999 ^b^	27.130 ± 0.929 ^c^	27.2670 ± 0.202 ^b^
Cell control	22.547 ± 0.855 ^c^	23.603 ± 0.762 ^d^	21.990 ± 0.743 ^c^

^a–d^ Data within a column without the same superscripts differ significantly (*p* < 0.05).
